# Mad from Life's History

**Published:** 1891-07-11

**Authors:** 


### The Doctor's Armchair.

### "MAD FROM LIFE'S HISTORY."

"Has she any children?" asked a metropolitan magistrate the other day of a constable who had charged a woman with an attempt to commit suicide. "She has had thirteen," said the constable, although it had been stated in the charge sheet that she was only thirty years of age. The woman was Eliza Drake, and she looked very delicate. She had jumped into the New River about mid-day, and would have been drowned if a kind-hearted postman, who happened to be passing, had not waded into the water and pulled her out. She had twice attempted to drown herself. The second time she was hastening into the deepest water she could reach, and would inevitably have succeeded in destroying herself but for the timely presence of her rescuer. The constable stated that the woman had been married very young. As for her husband, although he knew of her present trouble, he had not thought it necessary to stand by her when she was called upon to answer the grave charge of attempting to take her own life.

Eliza Drake was but a little past her girlhood, and she had had thirteen children. It is probable that she had married and commenced child-bearing at sixteen or seventeen. What does it mean for a young girl to be prematurely married and to bear a child every year for thirteen or fourteen years? Let any doctor, let any experienced mother, consider this question. The woman had probably been poorly fed; it is pretty certain also that she had been badly lodged. She was undoubtedly bloodless, for she looked delicate, said the report.

Her limbs were bloodless, and had no strength for work; her stomach was bloodless, and had little relish for food, and perhaps still less power of digestion; her brain was bloodless, and could not therefore think, or be patient, or avoid being irritable, impulsive, and passionate. Most probably she could not sleep. All this was inevitable from her physical condition. But there must be added to it the care of all her children. How many of the thirteen were still living? If only three or four, the care of them, in a small house and with little means, would be far more than her strength was equal to; but if seven or eight or ten demanded her unceasing watchfulness—what a task for a young creature, destitute of strength, of vitality and of blood.

The woman went mad; not permanently and hereditarily mad like a person born to insanity; but desperately, tragically, uncontrollably mad. The brain was so starved for want of blood, and so driven by the incessant calls and worries of daily and nightly duty, that it fell helpless, and despairing, like a horse that is galloped until it can gallop no more.

It is impossible to describe in words the pity that a physiologist feels for a woman thus driven to despair. No hell that any poet or prophet has described, can be more unendurable than this poor creature's daily lot had been. Hood almost broke his own heart, as well as the hearts of his readers with the "Song of the Shirt." How fitting are his words to describe the case of poor Eliza Drake:—

> "Mad from life's history;
> Glad to death's mystery;
> Swift to be hurled;
> Any where, any where, out of the world."

Every true physician, whose experience is large, and whose feelings are at all strong and deep, would pay fortunes for the gift of the poet, or for the power of the great prose writer. It is more than strange that the medical profession has given to the world no great poet. One might think, too, that some medical men would strive to utter their experience on canvas; would become painters in order to make the world see and understand what is daily taking place in its midst. But no physician or surgeon has ever been a great poet or painter, or even a novelist of more than average gifts. It is nevertheless still true that for the poet, the painter, and the novelist there are unworked fields of tragedy in the lives of poor women and children which would equal in pathetic interest the most tragic of the historic tragedies of the past. A poet, or even a great prose writer, with the training of a physiologist, and the experience of a physician might add records to the literature of human agony and endurance which would never die.

There is a practical moral to be added to the story of Eliza Drake. According to the law of this country males of fourteen and females of twelve, not subject to any physical or mental incapacity, are permitted to contract marriage. To both boys and girls this is a most dangerous permission—to girls it may prove a shocking cruelty. We, of the present time, do not seem to recognise that we have gained an eminence of knowledge and of scientific capacity which our forefathers did not possess. That eminence carries its corresponding responsibility. Physiology has taught us that the function of child-bearing should not be active until body and mind have reached maturity. When mere boys and girls marry—by boys and girls we mean persons of less than twenty-one or twenty-two years of age—they injure themselves both in mind and body. If, as generally happens, children are born to them at this immature period of life, the offspring thus born inherit the physical immaturity and the mental crudity of their parents.

Evils in crowds may and do result from the premature marriages which the law permits. Large numbers of the boys and girls born of such marriages in the poorer districts of populous towns have sickly little bodies and feeble and inconsequent brains. Not one in a hundred of such children ever becomes [a] thinker. The boys do not grow up strong enough for any of the manual occupations. They are not fit to be policemen or postmen, porters, or ploughmen; much less soldiers or sailors. They are not worthy of the name of Briton; they have not one of the true British characteristics; they might belong to any race, except that it would be difficult to find a race that would not be insulted by comparison with such wretched human waste and rubbish. Somebody has said that the lowest classes in our towns "breed like rabbits," but that they are of less value to the state than rabbits because they insist upon eating, whilst they cannot themselves be eaten.

We must face the fact that we are now breeding every year in this country hundreds of thousands of persons who are not worth breeding; persons who are a nuisance to themselves, a worry to the policeman and the magistrate, an expense to the taxpayer, and generally injurious all round. It would be a vast saving of energy and cost if such people were not born. For the birth of many of them the State itself is directly responsible by its permission of early marriages. But when the law which permits such very early marriages was made, physiology as a science hardly existed. Legislators did not know in any practical way the harm they were doing. But they do know now: we all know; we know as certainly as reason and truth can make us know that early marriages are ruinous to the common people; and we are fools to permit them to take place. There are hundreds of members of Parliament anxiously looking for a specialty: here is one for them. The man who shall effectually control the marriage market, and keep the breeding of immature children within manageable limits, will deserve a dukedom. The great invasion which this country has to fear at the present time is not an invasion of foreign troops, but of immature, sickly, useless, and superfluous babies.

